# Development of an HSV-1 production process involving serum-reduced culturing and bead-to-bead transfer

**DOI:** 10.1007/s00253-024-13193-4

**Published:** 2024-06-19

**Authors:** Helin Wang, Xiuhua Hu, Mingfang Zhang, Lin Yang, Yueying Xu, Xiaoxu Gu, Junjun Jiang, Weiwei Hu

**Affiliations:** Microbial and Viral Platforms (MVP), WuXi Biologics, 291 Fucheng Road, Hangzhou, 311106 China

**Keywords:** Herpes simplex virus type 1, Vero cells, Microcarriers, Bead-to-bead transfer, Serum-reduced culturing, Stirred-tank bioreactor

## Abstract

**Abstract:**

Herpes simplex virus type 1 (HSV-1) plays an important role in the field of gene therapy and viral vaccines, especially as an oncolytic virus. However, the mass production of HSV-1 viral vectors remains a challenge in the industry. In this study, a microcarrier-mediated serum-reduced medium culture was used to improve the bioprocess of HSV-1 production and increase HSV-1 yields. The composition of the culture media, which included a basal medium, serum concentration, and glutamine additive, was optimized. The process was successfully conducted in a 1 L bioreactor, and virus production was threefold greater than that of conventional processes with a 10% serum medium. The bead-to-bead transfer process was also developed to further increase scalability. In spinner flasks, the detachment rate increased from 49.4 to 80.6% when combined agitation was performed during digestion; the overall recovery proportion increased from 37.9 to 71.1% after the operational steps were optimized. Specifically, microcarrier loss was reduced during aspiration and transfer, and microcarriers and detached cells were separated with filters. Comparable cell growth was achieved with the baseline process using 2D culture as the inoculum by exchanging the subculture medium. To increase virus production after bead-to-bead transfer, critical parameters, including shear stress during digestion, TrypLE and EDTA concentrations in the subculture, and the CCI, were identified from 47 parameters via correlation analysis and principal component analysis. The optimized bead-to-bead transfer process achieved an average of 90.4% overall recovery and comparable virus production compared to that of the baseline process. This study is the first to report the optimization of HSV-1 production in Vero cells cultured on microcarriers in serum-reduced medium after bead-to-bead transfer.

**Key points:**

• *An HSV-1 production process was developed that involves culturing in serum-reduced medium, and this process achieved threefold greater virus production than that of traditional processes*.

• *An indirect bead-to-bead transfer process was developed with over 90% recovery yield in bioreactors*.

• *HSV-1 production after bead-to-bead transfer was optimized and was comparable to that achieved with 2D culture as inoculum*.

**Supplementary Information:**

The online version contains supplementary material available at 10.1007/s00253-024-13193-4.

## Introduction

Cancer remains a great threat to millions of people worldwide, and oncolytic virotherapy has shown promising results for the last two decades (Bell and McFadden 2015). Oncolytic viruses (OVs) preferentially replicate in tumor cells, induce immunogenic cell death, and stimulate host antitumor immunity (Kaufman et al. 2016). To date, four OVs have been approved for the treatment of advanced cancers (Shalhout et al. 2023), two of which are herpes simplex virus type 1 (HSV-1). Additionally, among the 97 ongoing OV studies, 23.7% are based on HSV-1 (Macedo et al. 2020). Due to the role of HSV-1 in the development of oncolytic viruses, the yield of oncolytic HSV-1 generated during production should be increased to meet industrial demand.

Due to the adherent characteristic of Vero cells, the optimization strategies for virus production based on Vero cell culture can be divided into two types. One strategy is to increase the virus yield per unit area. This can be achieved by optimizing the key process parameters of cell culture and virus production, including the medium, inoculation density, CCI, and harvest time (Kiesslich and Kamen 2020). The other method is to increase the culture area, which can be achieved by culturing with microcarriers in large-scale bioreactors. However, a large quantity of cells is needed to inoculate these bioreactors, which is difficult to achieve with static culture technology. Therefore, bead-to-bead transfer technology is necessary for the seed train (Yang et al. 2019).

Cell culture media for Vero cells, as a key factor that impacts cell growth and virus production, has attracted much attention, and a variety of media and supplements have been designed (Kiesslich and Kamen 2020). One key ingredient, FBS, is commonly used in culture media and provides essential nutrients and beneficial factors to improve cell growth; however, the addition of FBS may lead to challenges concerning lot-to-lot variation, animal welfare, supply, cost, and potential regulatory restrictions in the future. Thus, a serum-free cell culture process would be ideal for producing biologics. However, some challenges must be overcome to achieve serum-free culturing. The adaptation of cells to a serum-free medium may be time-consuming, and cells are sensitive to environmental stresses, such as shear and aeration stresses (Chisti 2000; dos Santos et al. 2011), which could limit their application. To balance virus production and serum usage, a variety of media with different concentrations of FBS were examined in this study, and a serum-reduced cell culture and virus production process was developed.

The application of microcarrier technology has greatly improved the ability to culture anchorage-dependent cells on a large scale. Microcarriers are small spheres with an approximate diameter of 100–300 microns (Chen et al. 2020). The microcarriers provide an attachment surface for anchorage-dependent cell growth. Compared to traditional static culturing methods, such as T-flasks and multilayers, the microcarrier technique greatly increases the area-to-volume ratio and reduces the process complexity, labor intensity, and cost of goods, especially in large-scale culture processes (Maartens et al. 2017).

On the other hand, large-scale culturing with microcarriers is currently limited by technical challenges, as bioreactors must be scaled up and bead-to-bead transfer must be efficient for harvesting cells and inoculating large-scale bioreactors. At present, scaling-out methods are widely used as alternatives to scaling-up methods; that is, cells cultured in multiple static systems are directly inoculated into multiple bioreactors, usually at a scale of approximately 10–50 L. The scaling-out approach could greatly simplify the scaling-up process but involves limitations regarding the large-scale production of viral vectors. Batch-to-batch variations introduced by the scaling-out approach may cause serious limitations, especially for drugs that have entered clinical phase III or received approval on the market. Additionally, the production scale is limited by the production facility, and the cost of labor and goods is higher because more bioreactors are operated simultaneously (Simaria et al. 2014).

In both scenarios (scale-up or scale-out), microcarrier bead-to-bead transfer is favored by the industry, and sophisticated control steps should be included during this process. Cells should be detached and harvested from microcarriers after combined treatment with dissociation reagents and shear force, which could also have negative effects on cell viability postharvest, growth, and virus production in subculture. The main challenge of the bead-to-bead transfer technique is to attain a high cell recovery ratio with acceptable cell conditions for subsequent culture and production. In the work reported here, the processes of microcarrier bead-to-bead transfer, subsequent cell, culture and virus production were established in small-scale bioreactors, which is a critical procedure for future large-scale production.

## Materials and methods

### Cell line, virus, and reagents

Vero cells (ATCC, CCL-81) at passages 129–150 were used in all experiments. Serum culture was obtained with DMEM (Thermo Fisher Scientific, USA) supplemented with 10% FBS (fetal bovine serum, Thermo Fisher Scientific, USA). Five commercial serum-free media, including OptiPro (Thermo Fisher Scientific, USA), were tested for serum-free and serum-reduced culturing, including OptiPro (Thermo Fisher Scientific, USA; designated as BM1 in subsequent sections), Vaccine (HyClone, Thermo Fisher Scientific, USA; designated as BM2), VP-SFM (Thermo Fisher Scientific, USA; designated as BM3), Nutri (Biological Industries, Sartorius, Germany; designated as BM4), and TransVero (Duoning Biotechnology, China; designated as BM5). For cell passage, 1 × TrypLE Express (Thermo Fisher Scientific, USA) and 10 × TrypLE Select (Thermo Fisher Scientific, USA) were used for cell detachment in static and microcarrier culture.

The virus seed of herpes simplex virus type 1 (HSV-1) was generated in-house. The original sequence accession number was NC_001806.2. The ICP34.5 gene was deleted, and the EGFP gene was inserted as an expression indicator. Viral titers were estimated with a standard plaque assay as previously described (Huleihel et al. 2001), except that medium containing carboxymethylcellulose sodium was used as an overlay, and 0.1% glutaraldehyde was used for fixation. To minimize batch-to-batch variations in virus titer measurement, HSV-1 produced from Vero cells cultured with DMEM supplemented with 10% FBS in CF1 was used as an internal control in all batches. The normalized sample titers were calculated as follows: the measured sample titer was divided by the internal control titer measured simultaneously, and the resulting ratio was multiplied by the average internal control titer of all related tests. The normalized cell-specific titers were calculated as follows: the measured sample titer was divided by the CCI to calculate the cell-specific titer, and then the cell-specific titers of all samples were divided by the cell-specific titer of the sample indicated in the figure legend to calculate the normalized cell-specific titer.

### Microcarrier preparation

Cytodex-1 (Cytiva Life Sciences, Sweden) was used in this study. Microcarriers were prepared and sterilized according to the manufacturer’s instructions. Briefly, all microcarriers were hydrated in Ca^2+−^ and Mg^2+^-free Dulbecco’s phosphate-buffered saline (DPBS), washed twice with fresh DPBS, autoclaved, and equilibrated in culture medium for at least 12 h before use in microcarrier culture experiments. For all microcarrier cultures, a microcarrier density of 3 g/L (culturing area of 1.32 × 10^4^ cm^2^/L) was used.

### Cell culture in T-flasks and multilayer flasks

The cells were seeded into 75 cm^2^ T-flasks or multilayer flasks (CF1/CF10; Thermo Fisher Scientific, USA) at an initial cell density of 1.5–2.0 × 10^4^ cells/cm^2^ for 3–4 days of culture and were grown to 80–90% confluence. For passaging, the cells were washed with DPBS and incubated for 8–10 min with 1X TrypLE at 37 °C.

### Cell growth and virus production in spinner flasks and bioreactors

#### Cell growth and virus production in spinner flasks

For all the spinner flask experiments, 125 mL spinner flasks were used. In the cell expansion stage, the working volume was 120 mL, and a 75 mL working volume was used in the virus production stage. The spinner flasks were placed on Micro-Stir Slow Speed Magnetic Stirrers (Wheaton, DWK Life Sciences, England) at agitation speeds ranging from 40 to 50 rpm in a 37 °C incubator with 5% CO_2_. The seeding density was 2 ± 1 × 10^4^ cells/cm^2^. The optional transfer VCD and CCI were 1.0–2.0 × 10^5^ cells/cm^2^. The temperature was 37.0 °C before virus infection and then decreased to 35.0 °C during the virus production stage. Seventy percent of the spent medium was exchanged with fresh medium to prevent nutrient limitation when the glucose concentration was lower than 2 g/L during the cell amplification phase before viral infection. For virus infection, cells grown on microcarriers within the target CCI range were allowed to settle, and 70% of the medium was exchanged with mixed virus seed and serum-free media (DMEM + 4 mM glutamine for the serum-containing process and VP-SFM + 4 mM glutamine for the serum-reducing process) was conducted. The MOI for virus infection was 0.01. The virus was harvested at 60–72 hpi (hours post-infection).

#### Determination of a suitable agitation speed for a homogeneous mixing in bioreactors

Upon insufficient agitation, pH and nutrient gradients may be serious problems for cell culture in a bioreactor, and microcarriers may settle or form an inhomogeneous distribution; however, excessive mixing may lead to high shear stress and microcarrier collisions, thus having a negative effect on process performance (Grein et al. 2019). Therefore, it is important to determine a suitable agitation speed for the microcarrier process in bioreactors. In this study, the agitation speed was selected as the minimal speed that delivers the highest uniformity of microcarriers, similar to previous studies (Yang et al. 2019). Briefly, 600 mL of 3 g/L Cytodex-1 in a 1 L bioreactor was agitated at candidate agitation speeds for at least 20 min and then sampled for microcarrier counting with a hemocytometer under a microscope. The number of microcarriers was divided by the theoretical volumetric microcarrier count (1.29 × 10^7^ mL^−1^) to calculate the uniformity.

#### Cell growth and virus production in bioreactors

Applikon Bio 1 L and 3 L multiuse glass autoclavable bioreactors (Getinge AB, Sweden) were used for cell culture and virus production. In bioreactor systems, the culture was agitated at 70–90 rpm in a 1 L bioreactor and at 50–70 rpm in a 3 L bioreactor. For culturing, 600 mL was used in a 1 L bioreactor, and 1.8 L was used in a 3 L bioreactor. At all bioreactor scales, the DO concentration was maintained at 40% by oxygen aeration, and the pH was maintained at 7.15 through CO_2_ gas with an upper limit of 7.40. The operation ranges of the seeding density, transfer VCD, MOI, and CCI, and the operations of virus infection and harvest were identical to those in the spinner flasks. Biomass monitoring was carried out at intervals of 30 s/2 min by measuring the online permittivity at 1000/10,000 kHz with Incyte Arc (Hamilton, Hoechst im Odenwald, Germany) or at 580 kHz/15,650 kHz with Futura (Aber Instruments, Aberystwyth, UK) dielectric probes. As the resulting capacitance is proportional to the total cellular membrane-bound volume, linear regression between the VCD measured offline and the corresponding online capacitance was performed, and the resulting formula was used to estimate the real-time VCD from the capacitance, as applied in previous studies (Fernandes et al. 2019; Moore et al. 2019; Surowiec and Scholz 2023) (Ref. Incyte Arc website: https://www.hamiltoncompany.com/process-analytics/cell-density-knowledge/cell-density-measurement-guidelines, Aber Futura website: https://aberinstruments.com/biotech/futura-probes/).

### Cell detachment on microcarriers and bead-to-bead transfer

Once confluence was reached, the microcarriers were allowed to settle, and the supernatant was removed by decanting. The microcarriers were then rinsed several times with Ca^2+−^ and Mg^2+^-free DPBS and DPBS-EDTA solution. Then, a mixture of 1X and 10X TrypLE (prewarmed to 37 °C) was aseptically added to the washed culture to a final TrypLE concentration ranging from 1.4-2.4X. The total height of the digestion mixture was slightly greater than that of the upper edge of the vessel impellers for efficient agitation. The cell detachment process was performed at 37 °C at a constant low speed (45 rpm in a 125 mL spinner flask and 76 rpm in a 1 L bioreactor) agitation or a combination of low- and high-speed (78–151 rpm in a 125 mL spinner flask and 127 rpm in a 1 L bioreactor, respectively) agitations. Samples were taken at 5-min intervals to monitor cell detachment by microscopic observation. The digestion step was terminated when more than 80% of the cells were detached from the microcarriers. The cell and microcarrier mixture was either supplemented with culturing medium and filtered through a 100 μm filter (Cat. No. 431,752, Corning, NY, USA) to separate the microcarrier and detached cells or inoculated into a subculture directly without filtration or termination.

### Offline measurements

The levels of glucose, lactate, ammonium, glutamine, and glutamate were measured with a Cedex Bio Analyzer (F. Hoffmann-La Roche Ltd, Basel, Switzerland). Viable cell density (VCD) and viability (VIA) were measured with a NuceloCounter® NC-200 system (ChemoMetec A/S, Allerod, Denmark) or a Vi-cell XR analyzer (Beckman Coulter, California, USA). The pH, pCO_2_, Na^+^, and K^+^ were measured with a RAPIDLab® 348EX Blood Gas System (Siemens Healthcare GmbH, Erlangen, Germany). Osmolality was measured with an OsmoTECH PRO osmometer (Advanced Instruments, Massachusetts, USA). A microscope (CKX53, Olympus, Tokyo, Japan) was used for cell culture observations.

### Statistical methodology

The datasets were processed in Minitab 18.1 (Minitab LLC., Pennsylvania, USA), Excel 2021 (Microsoft, Washington, USA), and R 4.1.2 (R Foundation) software for statistical analysis.

Then, 47 process and performance parameters from 8 batches with the serum-containing medium were analyzed, as listed in Table S3, to investigate the bead-to-bead transfer and subsequent cell culture and virus production processes. For parameter classification, the operation parameters were parameters with levels that could be directly controlled by process designs. Among the parameters that could be directly measured with analytical equipment or calculated from measurement results, the parameters that could not be directly influenced by additional operations were designated performance parameters, and the parameters that could be directly influenced by additional operations were designated mixed parameters. In particular, cell viability data were analyzed via logistic regression rather than linear regression, as the data were clustered close to the 100% limit. To do this, the data were logit transformed as described in a previous study (Manahan et al. 2019) before analysis. Briefly, the data were “logit transformed,” and then correlation analysis and PCA were performed on the transformed data. The logit transformation was calculated with the following formula:

$$logit\left(x\right)=\text{ln}\left(\frac{x}{1-x}\right)$$

Primary correlation analysis was performed between the parameters and ratios of sample volumetric infectivity titer/internal control infectivity titer (mentioned as “normalized titer proportion” in the following contents). A secondary correlation analysis was performed between the process parameters and the performance parameters, which showed a significant correlation with the normalized volumetric titer. Parameters with correlation factors ≥ 0.4 were regarded as positively related, and those with correlation factors ≤ −0.4 were regarded as negatively related.

The replication times of each experiment are indicated in the “Results” section or the figure legends. For the bar charts, the averages were plotted, and the error bars indicate the standard deviations.

### Experimental design

For a summary of all the experiments conducted during the process development in this study, please refer to Table S1.

For an expansion flow chart describing the seed train and overall process procedures of the serum-containing baseline, serum-reduced baseline, and serum-reduced bead-to-bead transfer (B2B) processes, please refer to Figure S5.

## Results

### Cell culture medium and supplement screening

**Fig. 1:**
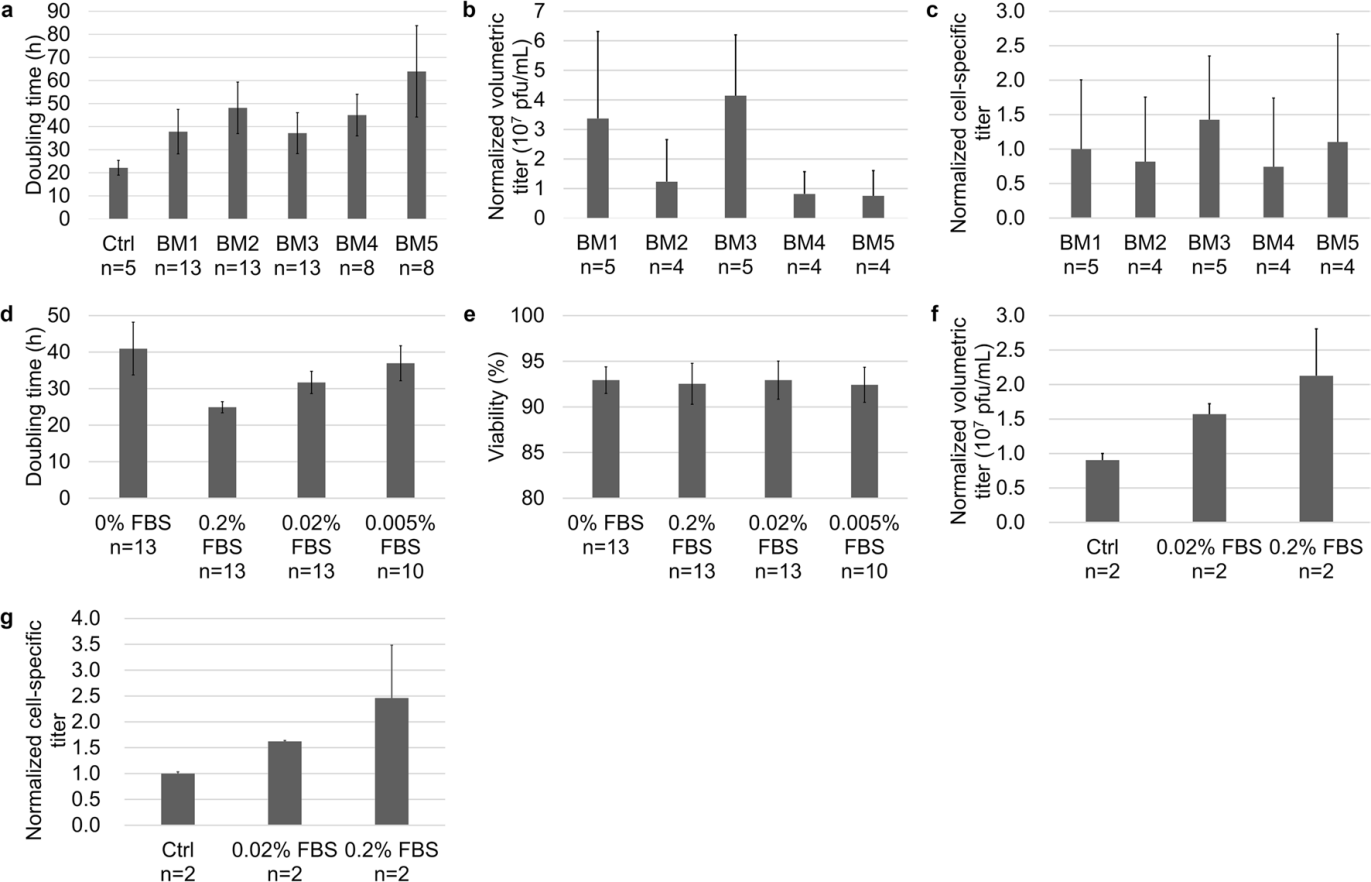
Performances of different basal media and supplementations. **a** Doubling times of Vero cells cultured in 5 types of serum-free media (BM1 - BM5). DMEM + 10% FBS was used as a control (Ctrl) (*n* ≥ 5). **b** Normalized volumetric titers of virus production in different serum-free media. **c** Cell-specific infectivity titers (normalized) of virus production in different serum-free media. All cell-specific infectivity titers were normalized to the average of BM1 (*n* ≥ 4). Doubling time (**d**) and viability (**e**) of Vero cells cultured in BM3 (VP-SFM) supplemented with different concentrations of FBS (*n* ≥ 10). Normalized volumetric titers (**f**) and cell-specific infectivity titers (normalized) (**g**) of virus production in BM3 with different FBS concentrations and DMEM + 10% FBS control. All cell-specific infectivity titers were normalized to the average of 0.2% FBS (*n* = 2)

To screen a suitable basal medium for the development of serum-reduced cell culture and virus production processes, five types of basal media (designated BM1-BM5) were tested for their ability to support cell growth and virus production. Among all tested serum-free media, BM3 (VP-SFM) had the shortest average doubling time (Fig. 1a) and the highest normalized volumetric titer (Fig. 1b) and cell-specific titer (Fig. 1c). Thus, VP-SFM was chosen for subsequent process development.

As residual serum was found to improve cell growth and virus production, the impact of different reduced levels of serum on cell growth and virus production was tested. Among all tested serum concentrations, the Vero cells directly adapted to and grew with the shortest doubling time in the VP-SFM supplemented with 0.2% v/v serum group (Fig. 1d), while the cell viability did not significantly differ among the different serum groups (Fig. 1e). Additionally, the normalized volumetric titers and cell-specific titers were highest in cells cultured with VP-SFM + 0.2% FBS (Fig. 1f, g). Therefore, VP-SFM supplemented with 0.2% FBS was used in our study. For glutamine supplementation, 4 mM glutamine instead of GlutaMax was chosen to decrease the doubling time and increase the viability of Vero cells (Figure S2 and S3).

### Optimization of microcarrier-mediated cell culture and virus production in bioreactors

#### Determining a suitable agitation speed in stirred-tank bioreactors

**Fig. 2:**
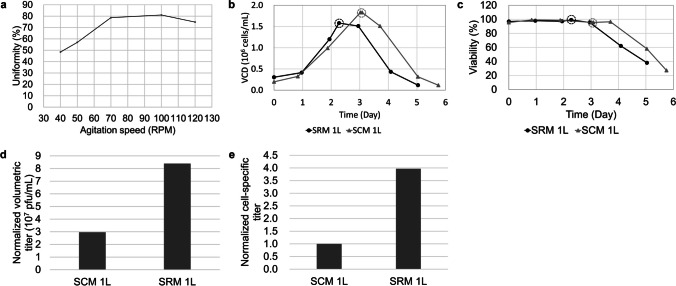
Development and performance of the HSV-1 production process in 1 L bioreactors. **a** Minimum agitation speed determination of a 1 L bioreactor for homogeneous microcarrier mixing. Vero cell growth curves (**b**) and viability curves (**c**) cultured with VP-SFM + 0.2% FBS medium (SRM 1 L) or DMEM + 10% FBS medium (SCM 1 L) in 1 L bioreactors. The times of infection are indicated with dashed circles. **d**,** e** Normalized volumetric titers and cell-specific infectivity titers (normalized) of virus production with DMEM + 10% FBS (SCM 1 L) and serum-reduced medium (SRM 1 L) in 1 L bioreactors are illustrated in **d** and **e**, respectively. All cell-specific infectivity titers were normalized to the value of SCM 1 L

To prevent gradient issues, microcarrier settling and shear stress damage to cells, proper agitation speeds and modifications to bioreactor setups should be applied during the microcarrier-related process. First, impeller scaling (ratio of impeller diameter/tank diameter) was maintained at approximately 0.47 at all scales, consistent with the impeller design of Thermo single-use bioprocess containers for microcarrier culturing (Thermo Scientific 2019). Second, the minimum agitation speed necessary for a sufficient microcarrier suspension, N_JS_ (JS for just suspended), was tested in 1 L bioreactors with a working volume of 600 mL. Microcarrier uniformity was measured as described in the “Results” section. Figure 2a shows that the uniformity of the microcarriers was maintained at the highest levels at agitation speeds ranging from 70 to 100 rpm. Therefore, 70 rpm was determined to be the minimum agitation speed for subsequent microcarrier culture in a 1 L bioreactor. For scaling-up, the equivalent power input per volume (P/V) was applied according to a previous study (Yang et al. 2019).

Moreover, other fluid dynamics parameters were also examined. Kolmogorov eddy length, calculated as described by Yang et al. (2019), was analyzed as a key parameter in microcarrier culture and decreased as agitation speed increased. The eddy length ranged from 157 to 163 µm at all studied scales, and the ratio of the Kolmogorov eddy length/microcarrier diameter ranged from 0.83 to 0.86, which is greater than the 2/3 ratio recommended by previous studies (Croughan et al. 1987) and likely causes no negative effects on cell culture with microcarriers. Additionally, the maximum shear stress was calculated as described in the literature (Merten 2015; Grein et al. 2019) and maintained at approximately 0.13 N/m^2^, which is close to the value recommended by the authors. For the calculation of the fluid dynamics parameters mentioned above, please refer to Table S2.

#### Vero cell culture and HSV-1 production in a 1 L bioreactor

For both serum-containing (SCM) and serum-reduced (SRM) cell culturing and virus production in 1 L bioreactors, operations were performed as described in “Cell growth and virus production in spinner flasks and bioreactors.” Sampling for virus titer measurement was conducted at certain HPI intervals. The profiles of cell growth and viability are given in Fig. 2b and c. The virus production levels are illustrated in Fig. 2d and e. Compared with the traditional serum-containing process, the serum-reducing process results in an approximately threefold increase in the virus titer.

### Vero cell bead-to-bead transfer studies in spinner flasks

As a large amount of oncolytic viral vector is needed for therapeutic use and the ability to provide enough seed cells for the inoculation of large-scale bioreactors by 2D culturing is limited, it is essential to culture seed cells with microcarriers and inoculate them at the production scale via the bead-to-bead transfer process. The preliminary bead-to-bead transfer experiments in this study were conducted in 125 mL spinner flasks as scale-down models. TrypLE was chosen as the cell dissociation reagent because it did not originate from animals and complied with cGMP manufacturing.

Once confluence or targeted VCD transfer was achieved, the microcarriers were washed with the procedure described in “Cell detachment on microcarriers and bead-to-bead transfer”. The 1X and 10X TrypLE reagents were mixed and added to the washed microcarrier cultures to final concentrations ranging from 1.4–2.4X. The final concentration was controlled to efficiently detach the cells and to prevent over-digestion, which led to severe microcarrier or cell aggregation and decreased viability. Moreover, the final serum concentration in the digestion mixture was calculated and controlled by the washing procedure so that residual serum would not counteract the activity of the dissociation reagent.

#### Cell harvest optimization

**Fig. 3:**
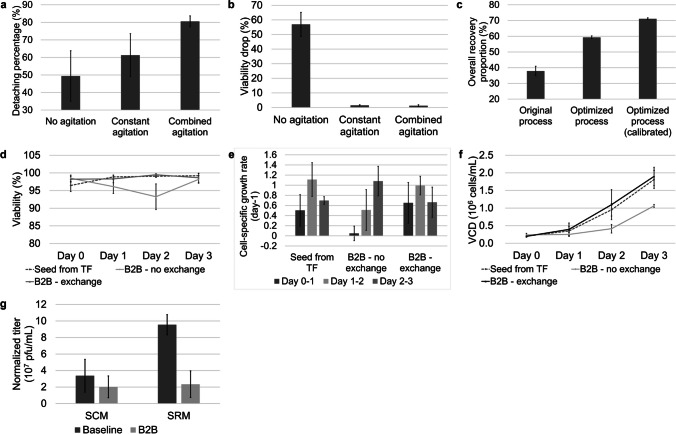
Preliminary development of the bead-to-bead transfer process. Detaching percentages (**a**) and viability drops (**b**) of the digestion process with no agitation, constant agitation, and combined agitation (*n* = 3). **c** Overall recovery proportions of the original, optimized, and optimized processes calibrated with sampling loss (*n* = 2). The calibration was calculated as described in Table S2. Cell viability curves (**d**), growth rate comparison (**e**), and cell growth curves (**f**) of (1) Vero cells cultured in T-flasks and transferred to microcarriers (seed from TF); (2) cells cultured with microcarriers, treated with a bead-to-bead transfer process and cultured without medium exchange after the attaching period (B2B - no exchange); and (3) cells cultured with microcarriers, treated with a bead-to-bead transfer process and cultured with medium exchange after the attaching period (B2B - exchange) (*n* = 4). **g** The normalized volumetric titer of virus production in cells subjected to the baseline process (baseline) or bead-to-bead transfer process (B2B), cultured in DMEM + 10%FBS (SCM) or VP-SFM + 0.2%FBS (SRM) media. (SCM baseline, *n* = 8; SCM B2B, *n* = 8; SRM baseline, *n* = 3; SRM B2B, *n* = 5)

The original digestion procedure was performed with intermittent gentle mixing as described in a previous publication (Yang et al. 2019). However, the percentage of detached cells was low (average 49.4%, Fig. 3a) even with extended incubation, and the viability of the cells decreased substantially (average viability decrease of 56.9%, Fig. 3b), which may be caused by differences in the dissociation reagents used. Therefore, constant agitation by spinner flasks placed on stirrers was applied. As shown in Fig. 3a and b, digestion with constant agitation increased the average detachment percentage by approximately 10% with a much shorter digestion time (approximately 40 min shorter). The decrease in viability after digestion decreased significantly, indicating that the digestion procedure caused little damage to the cells. To further increase the detachment percentage, short-term intense agitation was used according to related research (Nienow et al. 2016). The combined agitation led to an increase in the average detachment percentage to 80.6%, while an average viability decrease (1.2%) comparable to that of constant agitation was maintained.

Next, a series of optimizations were conducted to increase the yield of the digestion procedure, which included increasing the residue volume during the washing procedure, reducing the number of transfer operations during the whole process, and separating the cell and microcarrier with a filter instead of sediment. Additionally, the overall recovery proportions were calibrated with sampling loss, as described in Table S2, as sampling loss severely affected the final yield calculation on a small scale. The optimized process delivered an average calibrated overall recovery proportion of 71.1%, which was much greater than that of the original process, with an average overall recovery proportion of 37.9% (Fig. 3c).

#### Cell culturing optimization and virus production

The results showed that in the bead-to-bead transfer subculture, cell viability decreased from Day 0 to Day 2 (Fig. 3d, B2B - no exchange), and the cell-specific growth rate greatly decreased, especially from Days 0–1 (Fig. 3e). The medium exchange was observed to improve the cell growth in subculture after the bead-to-bead transfer process, indicating that the retardation of cell growth was caused by the presence of residue dissociation reagent, which is consistent with previous reports (Rourou et al. 2013). Thus, an additional medium exchange of approximately 50% after the attachment period (approximately 4–8 h) of subculture was applied; the microcarriers were allowed to settle, the supernatant was removed, and fresh medium was added to the reduced residue dissociated reagents from the culture. With medium exchange after the attachment period, the cell viability and cell growth were recovered and comparable to those of the T-flasks sub-cultured with seeds (Fig. 3d–f, B2B - exchange). However, the amount of virus produced by bead-to-bead transfer (B2B) cells was much lower than that produced by the baseline process with seeds from traditional 2D culturing in both the SCM and SRM culturing systems (Fig. 3g); thus, the process should be further optimized.

### Optimization of the bead-to-bead transfer process for virus production

As virus production was negatively affected by the bead-to-bead transfer process, both in serum-containing and serum-reduced processes, the combination of bead-to-bead transfer, subsequent cell culture, and virus production processes was optimized. The process parameters that significantly affected virus production were identified via correlation analysis. Additionally, principal component analysis (PCA) was conducted to enrich and cluster the critical process parameters that needed to be optimized.

#### Data preparation

As more historical data were available for the serum-containing process, they were selected as the data source for analysis. Forty-seven parameters (listed in Table S3) and infectivity titers of 8 batches from previous experiments were collected and analyzed. The parameters were classified into operation, performance, and mixed parameters as described in “Statistical methodology.”

In particular, as the viability data were expressed as percentages and clustered close to the 100% limit, they were analyzed via logistic regression as described in the “Statistical methodology.”

#### Primary and secondary correlation analysis

**Fig. 4:**
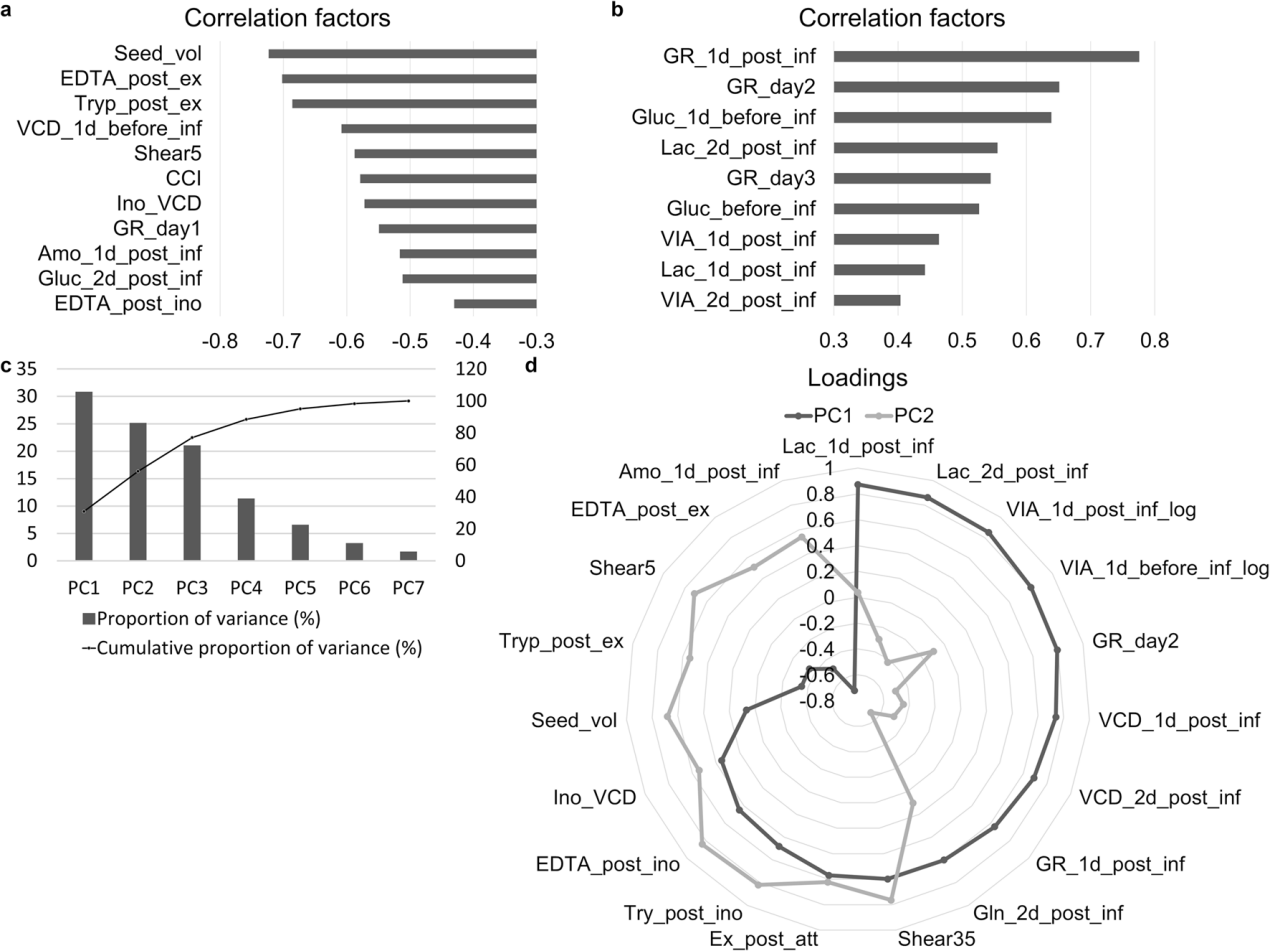
Virus production optimization of bead-to-bead and subsequent processes. **a**,** b** Correlations between operation and performance parameters and normalized volumetric titers were calculated. Parameters with correlation factors ≤−0.4 and ≥ 0.4 are shown in **a** and **b**, respectively. **c** Proportion of variances of the principal components determined with PCA. **d** Loadings of principal components 1 and 2. The loadings of the top 10 parameters of the two principal components are plotted

For primary correlation analysis, correlations between all parameters and the normalized volumetric titers were calculated, and parameters with correlation factors ≤−0.4 or ≥ 0.4 were regarded as negatively or positively related, respectively, as shown in Fig. 4a and b. The following process parameters were negatively related to virus production: concentration of EDTA (EDTA_post_ex) and TrypLE (Tryp_post_ex) after the medium exchange, shear stress during the last 5 min of digestion (Shear5), CCI, and inoculation density (Ino_VCD).

As several performance parameters were significantly related to virus production and could be controlled only through process parameters, a secondary correlation analysis between the process and performance parameters was conducted. To identify parameter for the secondary correlation analysis, performance parameters with positive or negative correlations with the normalized titer proportions were selected, and candidate process parameters for optimization were selected with the following criteria: (1) If the correlation between the process parameter and performance parameter was opposite/consistent to the correlation between the performance parameter and normalized titer proportions, the process parameter received one negative/positive mark, which was calculated as −1/+1, respectively. (2) The total marks of each parameter were calculated, and parameters with absolute values not lower than 4 were selected as candidate parameters for optimization. The selected process parameters were identical to those used in the primary analysis, which provided additional evidence for target parameter screening. The correlation factor matrix of the secondary correlation analysis and the marks of related process parameters are shown in Figure S1 and Table S4, respectively.

#### Principal component analysis

PCA was also used to identify significantly related parameters. The variance proportion and cumulative proportion of principal components (Fig. 4c) showed that the first (PC1) and second (PC2) principal components accounted for 56.0% of the variance. The loadings of the first ten parameters of PC1 and PC2 (Fig. 4d) showed that the loadings of PC1 are mostly performance parameters, and the loadings of PC2 are mostly process parameters. The following process parameters were highly related to virus production: shear stress from low- and high-speed agitation during digestion (Shear5 and Shear35), inoculation VCD (Ino_VCD), seed volume (Seed_vol), EDTA concentration after inoculation (EDTA_post_ex), medium exchange after the attaching period (EX_post_att), and TrypLE and EDTA concentration after medium exchange (Tryp_post_ex and EDTA_post_ex).

#### Process optimizations

Overall, the following optimizations were suggested based on analysis of historical data: (1) the concentrations of residual EDTA and TrypLE in the subculture were reduced, which was accomplished by additional media exchange after the attachment period; and (2) the shear stress during digestion was decreased. As a decrease in the agitation speed may lead to insufficient mixing during digestion, the speed of high-speed agitation decreased. (3) The cell densities of inoculation and infection during subculturing also decreased. As the effect of inoculation density may be a secondary effect that reflects the effect of the TrypLE residue and EDTA in subculture and the decrease in inoculation density may lead to an uneven distribution of cells between the microcarriers and the elongated lag phase, the effects of both the original and optimized inoculation densities were tested in subsequent experiments. The original and optimized levels (Table S5) of the selected parameters were determined by regression analysis (Figure S4).

#### Validation of the optimized process in spinner flasks

**Fig. 5:**
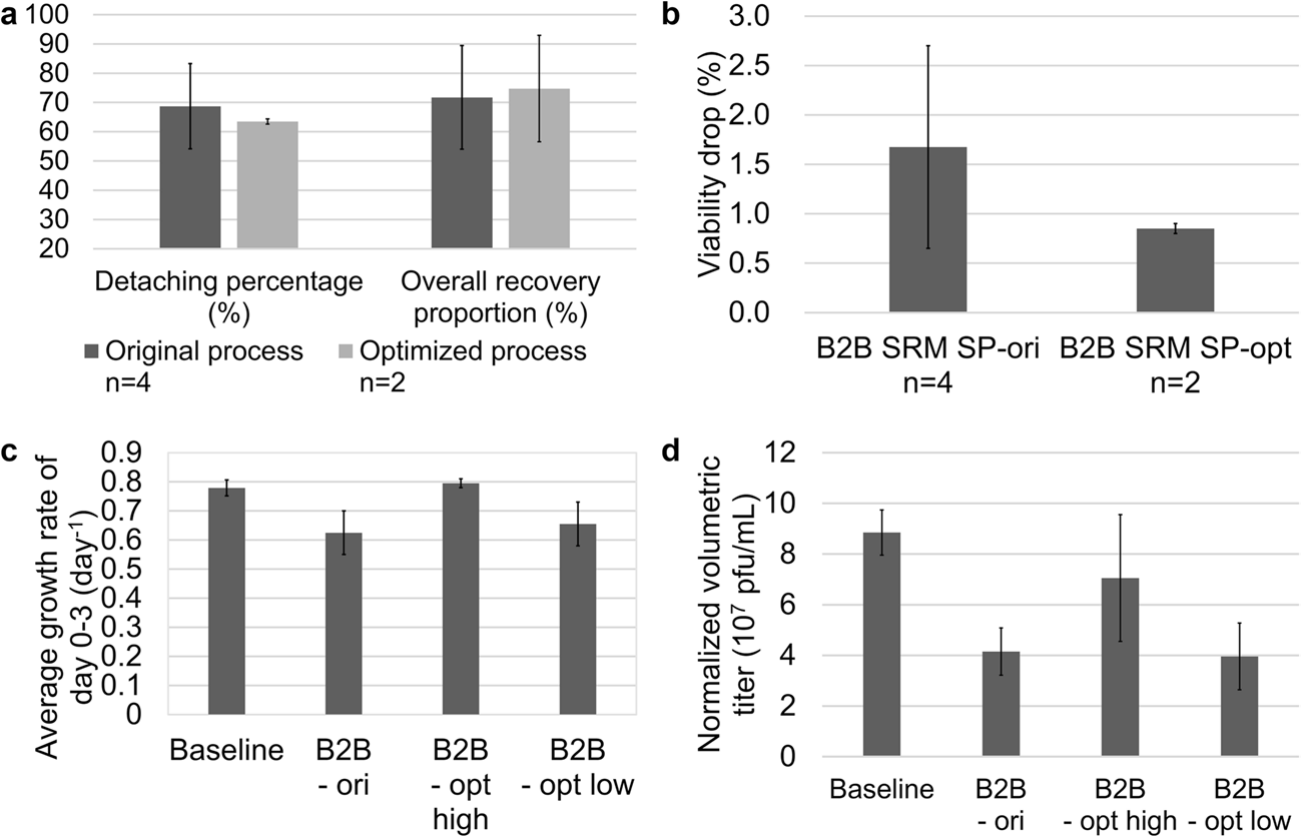
Performance of the optimized bead-to-bead transfer process with serum-reduced medium cultured in spinner flasks. Detaching percentage and overall recovery proportion (**a**) and viability drop (**b**) comparison of the original and optimized bead-to-bead transfer processes. Average growth rates of Day 0–3 (**c**) and normalized volumetric titers (**d**) in subcultures of original (B2B - ori), optimized with high inoculation density (B2B - opt high) and optimized with low inoculation density (B2B - opt low) bead-to-bead transfer processes

As the process with serum-reduced medium (VP-SFM + 0.2% FBS) performed better than that with serum-containing medium (DMEM + 10% FBS), subsequent process developments were conducted with the serum-reduced medium. The data of the baseline process with seeds from static culture were used as a positive control (“Baseline” in Fig. 5) for the validation. The data of the original bead-to-bead transfer process with all five process parameters (refer to Table S4) at the original levels were used as a negative control (“B2B - ori” in the figure). The optimized process was tested with the following variants: in one test, all five candidate process parameters were modified to the optimized level (B2B - opt low); in the other test, the parameters, except for inoculation density, were modified to the optimized level, while the inoculation density of the subculture was maintained at the original level, which was higher than the optimized level (B2B - opt high).

Compared with the original process, the optimized process at the bead-to-bead transfer stage resulted in a slightly decreased detachment percentage but a slightly increased overall recovery proportion (Fig. 5a), and the variation in the detachment percentage greatly decreased. Additionally, both the level and variation of the cell viability decrease were lower in the optimized process than in the original process (Fig. 5b).

For cell growth in subculture, the optimized process with a low inoculation density (B2B - opt low) had almost no beneficial effect on the cell growth rate. But when the original inoculation density was maintained, an increase in the cell growth rate was achieved, with a level comparable to that of the baseline process; in this process, cells from static culture were utilized as the inoculum (Fig. 5c).

The average virus titer was 7.0 × 10^7^ pfu/mL with the optimized process with high inoculation density (B2B - opt high), which was higher than the average titer of 4.1 × 10^7^ pfu/mL with the original process but lower than that of the baseline process (8.8 × 10^7^ pfu/mL), with a greater variation (Fig. 5d).

The phenomenon of greater variation may be caused by less well-controlled environmental parameters in the spinner flasks, especially shear stress and temperature, which may impact the performance of cells with high sensitivity after the cells are subjected to the bead-to-bead transfer process. Considering that bioreactors offer better control than that of spinner flasks, it was determined that the following processes would proceed in bioreactors.

### Vero cell bead-to-bead transfer and virus production in bioreactors

**Fig. 6:**
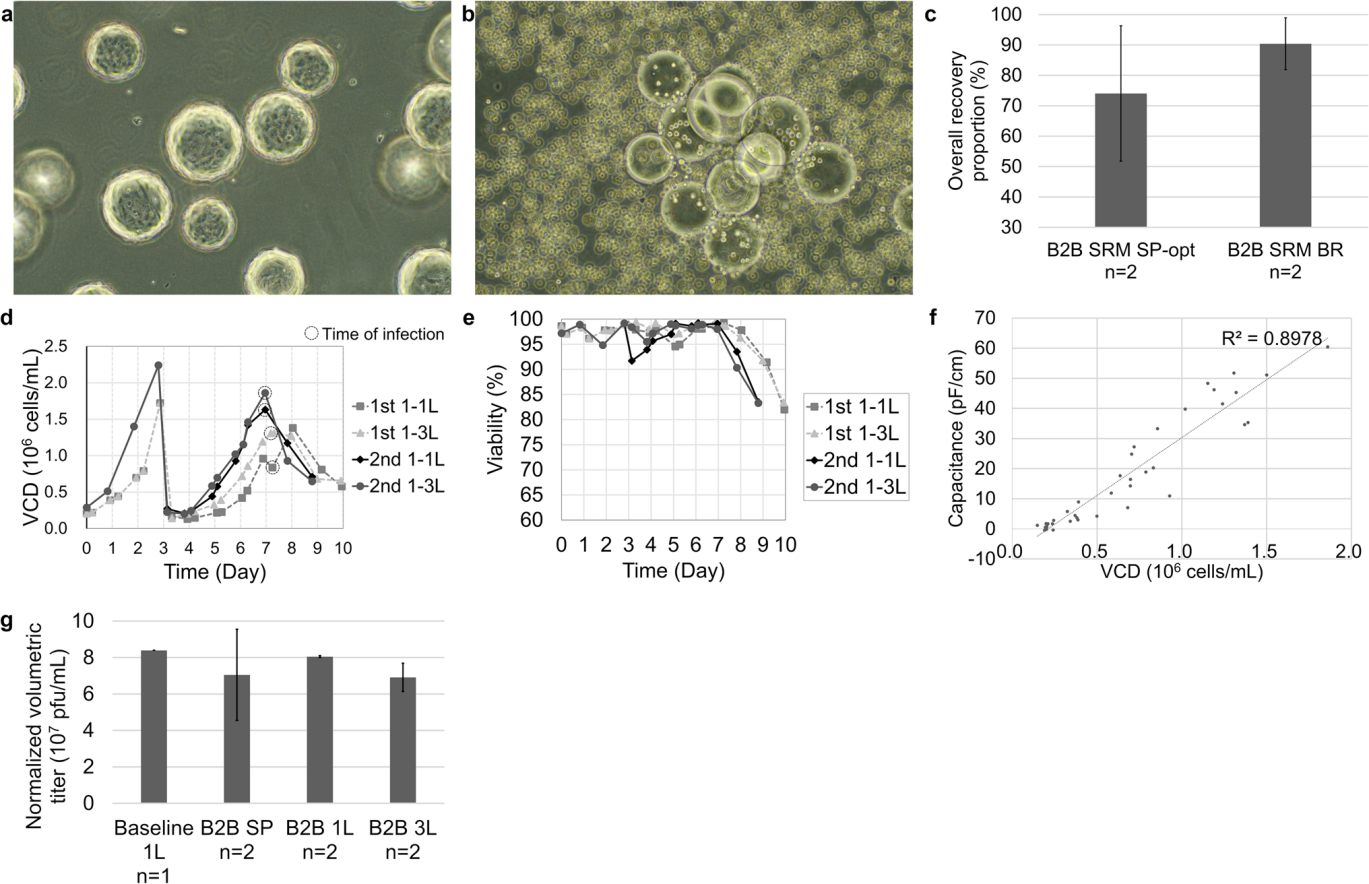
Technical transfer of the bead-to-bead transfer process to bioreactors. **a** Microscopy image of confluent Vero cells cultured with Cytodex-1 immediately before digestion (100×). **b** Microscopy image of the digested mixture showing Cytodex-1 with detached Vero cells (100×). **c** The overall recovery proportions of the optimized bead-to-bead transfer process conducted in spinner flasks (B2B SRM SP-opt) and bioreactors (B2B SRM BR) with serum-reduced medium. **d** Cell growth curve of seeds cultured in a 1 L bioreactor (Day 0–2.8) and sub-cultured and virus production in a 1 L and 3 L bioreactor (Day 3.8–8.8). The dashed circles indicate times of infection. **e** Viability curves. **f** Correlation between viable cell density measured by NC-200 and permittivity values measured by the Aber sensor. *R*^2^_adjs_ = 0.8950. **g** The normalized volumetric titers of the baseline serum-reducing process in a 1 L bioreactor (Baseline 1 L), the virus production process with seeds from optimized bead-to-bead transfer in a spinner flask (B2B SP), and the virus production process with seeds from bead-to-bead transfer in 1 L (B2B 1 L) and 3 L bioreactors (B2B 3 L)

The optimized process was conducted twice with a 1 L bioreactor as the N-1 stage and 1 L and 3 L bioreactors as the N stage. A 1 L bioreactor with 3 g/L Cytodex-1 was inoculated with Vero cells, and digestion was conducted after 3 days of culture when the VCD reached 1.2–1.7 × 10^5^ cells/cm^2^. Confluency was checked with a microscope, as shown in Fig. 6a. Cell digestion was conducted as described in “Cell detachment on microcarriers and bead-to-bead transfer.” After digestion, efficient detachment of the cells was confirmed via microscopic observation, as shown in Fig. 6b. From the two trials, average total viable cells of 7.33 × 10^8^ were obtained from the 1 L seed bioreactor. The digested mixture was inoculated into 1 L and 3 L bioreactors with 3 g/L Cytodex-1. The time of infection was comprehensively determined by both VCD measurements with NC-200 and the correlation between the offline VCD measurements and the online permittivity measurements (Fig. 6f). Harvesting was conducted 60–72 h after infection.

The overall recovery proportion of the bead-to-bead transfer processes conducted in bioreactors was approximately 90%, which was higher than that in spinner flasks with decreased variation, possibly because the operations were more precisely controlled (Fig. 6c). The cell growth and viability curves of both trials are shown in Fig. 6d and e. The difference in cell growth between the two trials was mainly caused by the difference in inoculation density, which was greater in the second trial than in the first trial.

For the N stage, the infection timepoints are indicated by dashed circles. For virus production, the CCIs of the 3 L bioreactor in the second trial and the 1 L and 3 L bioreactors were within the range of 0.99–1.11 × 10^5^ cells/cm^2^. For the 1 L bioreactor of the 1st trial, although the VCD measured for NC-200 was 0.63 × 10^5^ cells/cm^2^ before infection, the dielectric measurements showed that the VCD was nearly 0.99 × 10^5^ cells/cm^2^; thus, the cells were infected with HSV-1. As the process was better controlled in the bioreactors than in the spinner flasks, the normalized volumetric titers of the 1 L subculture bioreactors after the bead-to-bead transfer process (B2B 1 L) were greater than those of the experiments conducted in the spinner flasks (B2B SP) and comparable to those of the virus production experiment conducted in a 1 L bioreactor with seeds from 2D culturing (Baseline 1 L). Additionally, the standard deviations of virus production in the 1 L and 3 L bioreactors (B2B 1 L and B2B 3 L) were much lower than those in the spinner flasks, reflecting an increase in process robustness (Fig. 6g).

## Discussion

The development of vaccines, gene therapies, and cell therapies offers new hope for millions of people, but their application has increased the need for scalable and cost-effective production of cells and viruses. Due to the high area-to-volume ratio and semi-suspension culturing of microcarriers, the ability of microcarrier technology to support massive production is inherent (Merten 2015). Additionally, compared with traditional 2D culturing of anchorage-dependent cells, microcarrier culturing greatly reduces the cost of goods, labor, and space requirements (Simaria et al. 2014). Unfortunately, the scale of microcarrier-based production is limited by the adoption of an efficient bead-to-bead transfer process that utilizes microcarriers for inoculum culturing in the seed train (Yang et al. 2019). Additionally, there are no reports available concerning the effect of the bead-to-bead transfer process on subsequent virus production, which was addressed in this study.

Overall, our studies established a microcarrier-based process to culture Vero cells and produce HSV-1 with serum-reduced medium and microcarrier culture at the N-1 stage.

We optimized the type and composition of the serum-reduced medium, including the serum concentration and type of glutamine supplement. Compared to the production attained using a classical medium supplemented with 10% serum in bioreactors, a threefold greater virus production was achieved with VP-SFM + 0.2% FBS + 4 mM glutamine. Additionally, the serum concentration after virus infection was further reduced with 70% medium replacement with serum-free medium, thus facilitating downstream purification. Although Vero cell culture and virus production with serum-free media have been developed in recent years (Kiesslich et al. 2020; Sia et al. 2023), our data showed that virus production was higher with 0.2% serum. For future investigations aiming to fully remove serum from the medium while maintaining comparable virus production, additional commercial serum-free media and animal-origin-free supplements, such as recombinant growth factors, may be tested.

Bead-to-bead transfer is a critical step for the scale-up of microcarrier-based cell culture. Although direct bead-to-bead transfer through bridging of cells between confluent and fresh microcarriers has been reported for several cell lines (Chen et al. 2020), this process was not feasible for the Vero cells used in-house. Additionally, direct bead-to-bead transfer leads to a severely uneven distribution of cells between microcarriers, which has a negative effect on virus production (Merten 2015). In this study, an indirect bead-to-bead transfer process was developed for preparing an inoculum of Vero cells grown on Cytodex-1 microcarriers.

The detachment efficiency, overall recovery proportion, and cell growth in subculture were improved by optimizing the operational steps; to achieve these optimizations, agitation was combined during digestion, disturbance and transfer of the microcarrier slurry were decreased during washing and digestion, cell and microcarriers were separated with filters, and medium exchange was performed in subculture. To further improve virus production after the bead-to-bead transfer process, critical process parameters, including shear stress during digestion and CCI, were identified and optimized from historical data via correlation analysis and PCA. The amount of virus produced by the optimized process was 1.7-fold greater than that produced by the original process. The optimized bead-to-bead process was then applied in bioreactors. An average overall recovery of 90% was obtained in two replicates, and similar virus productions were obtained to those of baseline processes utilizing seeds from static culture, indicating that the virus production process with microcarrier culture at the N-1 stage was successfully established.

As regulatory agents encourage animal-origin reagents to be removed during the manufacturing of drug substances, animal-free reagents are favored for the bead-to-bead transfer process. Thus, TrypLE was selected as the dissociation reagent instead of trypsin. We found that the process was strongly impacted by the dissociation reagent selected. TrypLE is far more stable than trypsin at 37 °C (Nestler et al. 2004) and a mixture of high concentrations of TrypLE was used in the development process; thus, medium exchange was necessary to replace the residue dissociation reagent. This operation led to increased operation complexity and cost but can be avoided through inhibiting TrypLE activity by adding soybean trypsin inhibitor (STI) as a termination reagent to the proper concentration, according to a previous study (Rourou et al. 2013); however, we found that the commercially available low-concentration STI did not effectively terminate digestion (data not shown). On the other hand, the developed process does not rely on additional devices, such as a Thermo Harvestainer, which is usually used to separate microcarriers and cells (Ton et al. 2023). This led to simplified operations, reduced costs, and decreased space requirements.

The statistical analysis we performed on historical bead-to-bead transfer experimental data was roughly based on linear correlation (Bartholomew 2010), which is less precise than the methods used for structurally designed experiments with statistical methods, such as design of experiment (DoE) (Narayanan and Love 2022). This strategy may support future development works on early-stage process when cost and timeline requirements limit the application of complex DoE methods, and multiple parameters should be screened and optimized. On the other hand, compared to inoculating a bioreactor with seeds from static culturing, performing bead-to-bead transfer is more complex, and the process should be more accurately controlled. Further optimizations may be necessary during the technical transfer and scaling up to the manufacturing scale. The technique developed here shows great potential because it will enable large-scale virus production with microcarrier technique-based cell culturing, which is limited when only static culturing serves as a seed for this production stage.

In conclusion, the medium composition was optimized to increase virus production threefold and reduce the serum concentration to 0.2%; in addition, a microcarrier bead-to-bead transfer process with a 90% recovery rate was developed and virus production was optimized. This study provides further insight into the feasibility of applying microcarrier technology for large-scale cell culture and virus production applications for gene and cell therapy and for vaccine production.

## Supplementary Information

Below is the link to the electronic supplementary material.

Supplementary Material 1 (PDF 481 KB)

## Data Availability

The authors confirm that the data supporting the findings of this study are available within the article and its supplementary materials. For the list of abbreviations used in the article, please refer to Table S6.
