# Supplementary material for: Development of an HSV-1 production process involving serum-reduced culturing and bead-to-bead transfer
Source: Appl Microbiol Biotechnol. 2024 Jun 19;108:383. doi: 10.1007/s00253-024-13193-4 (PMC11186949; doi:10.1007/s00253-024-13193-4)
Supplement: Supplementary file 1 [file 253_2024_13193_MOESM1_ESM.pdf]

Supplementary information

Journal name: Applied Microbiology and Biotechnology

Manuscript title: Development of an HSV-1 production process involving serum-reduced culturing and bead-to-bead transfer

The names of the authors: Helin Wang[1], Xiuhua Hu[1], Mingfang Zhang[1], Lin Yang[1], Yueying Xu[1], Xiaoxu Gu[1], Junjun Jiang[1*], Weiwei Hu[1*]

The affiliations and addresses of the authors:

[1] Microbial and Viral Platforms (MVP), WuXi Biologics, 291 Fucheng Road, Hangzhou 311106, China

Corresponding author:

Dr. Weiwei Hu

E-mail: hu_weiwei0501@wuxibiologics.com

Dr. Junjun Jiang

E-mail: jiang_junjun@wuxibiologics.com

Telephone and fax number: +86-571-60954091

Supplementary figures:

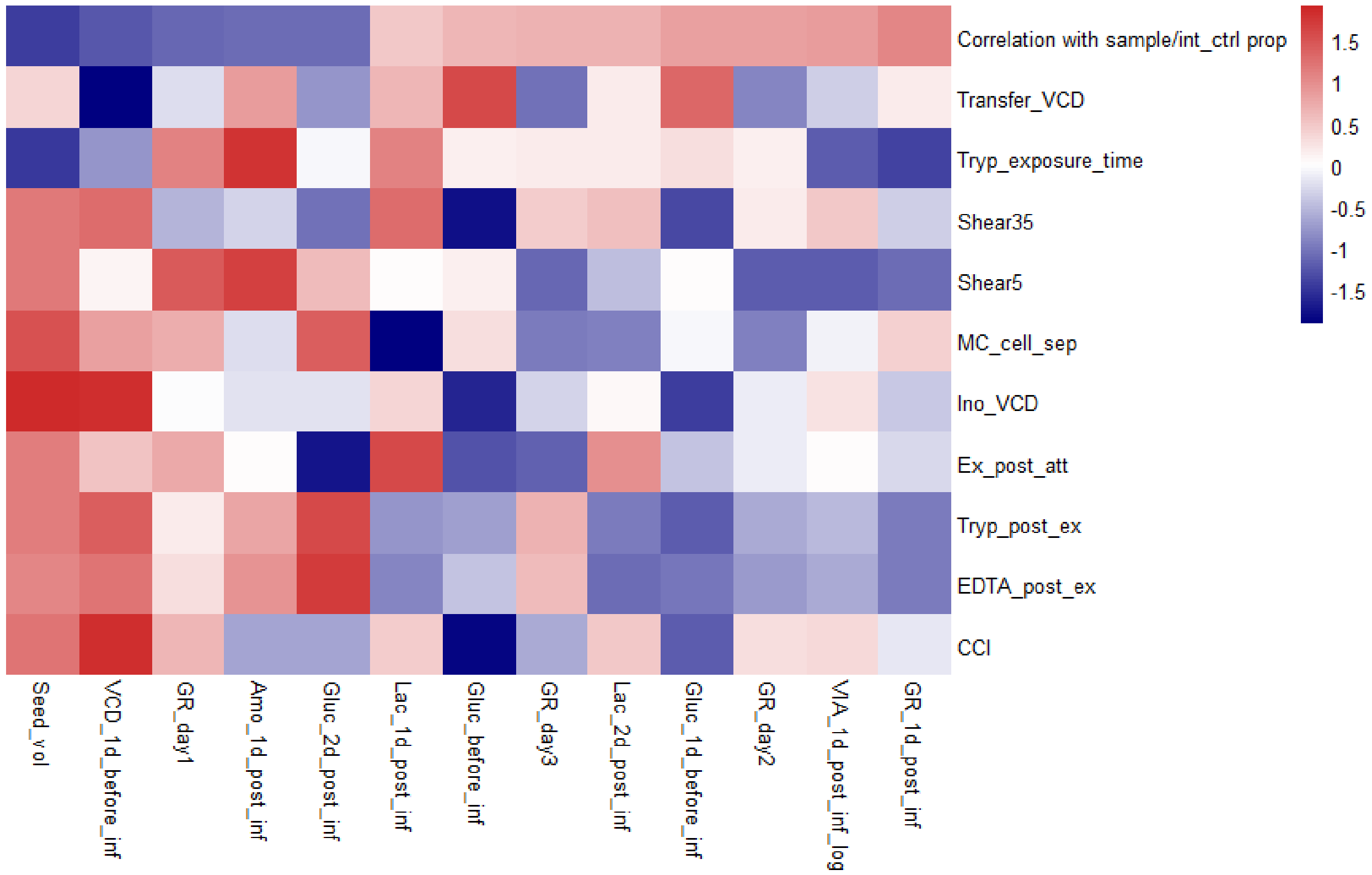

Figure S1 Correlation factor matrix of secondary correlation analysis

The first row of the matrix shows the correlation between the performance parameters and the sample/internal control proportion (normalized titer). The other rows show the correlation between the performance parameters and the process parameters.

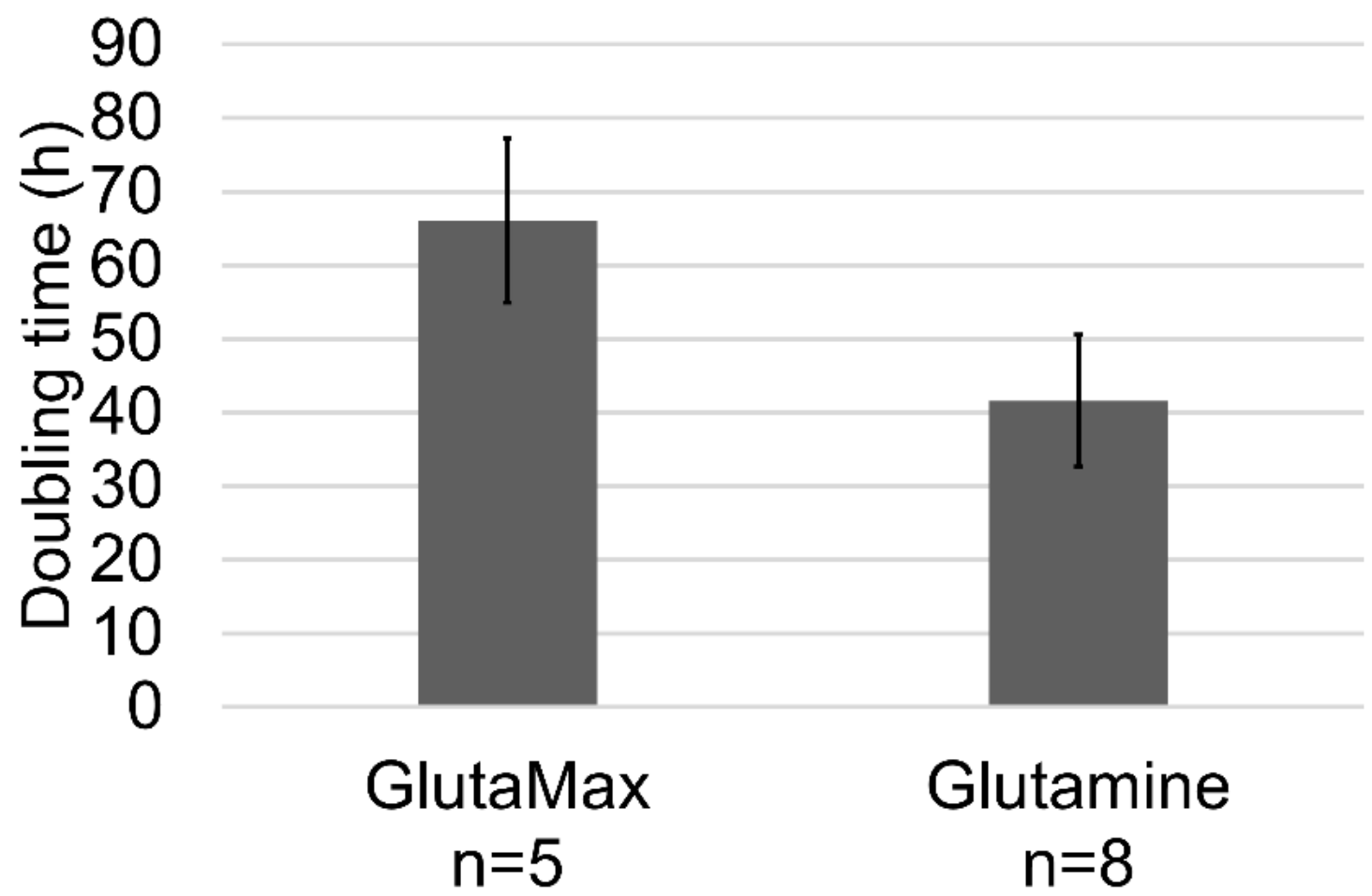

Figure S2 Doubling time of Vero cells cultured with GlutaMax or glutamine as supplements

Vero cells were cultured with VP-SFM medium in T flasks, and GlutaMax (n=5) or glutamine (n=8) were used as source of glutamine. Doubling times of the cells were shown in the figure.

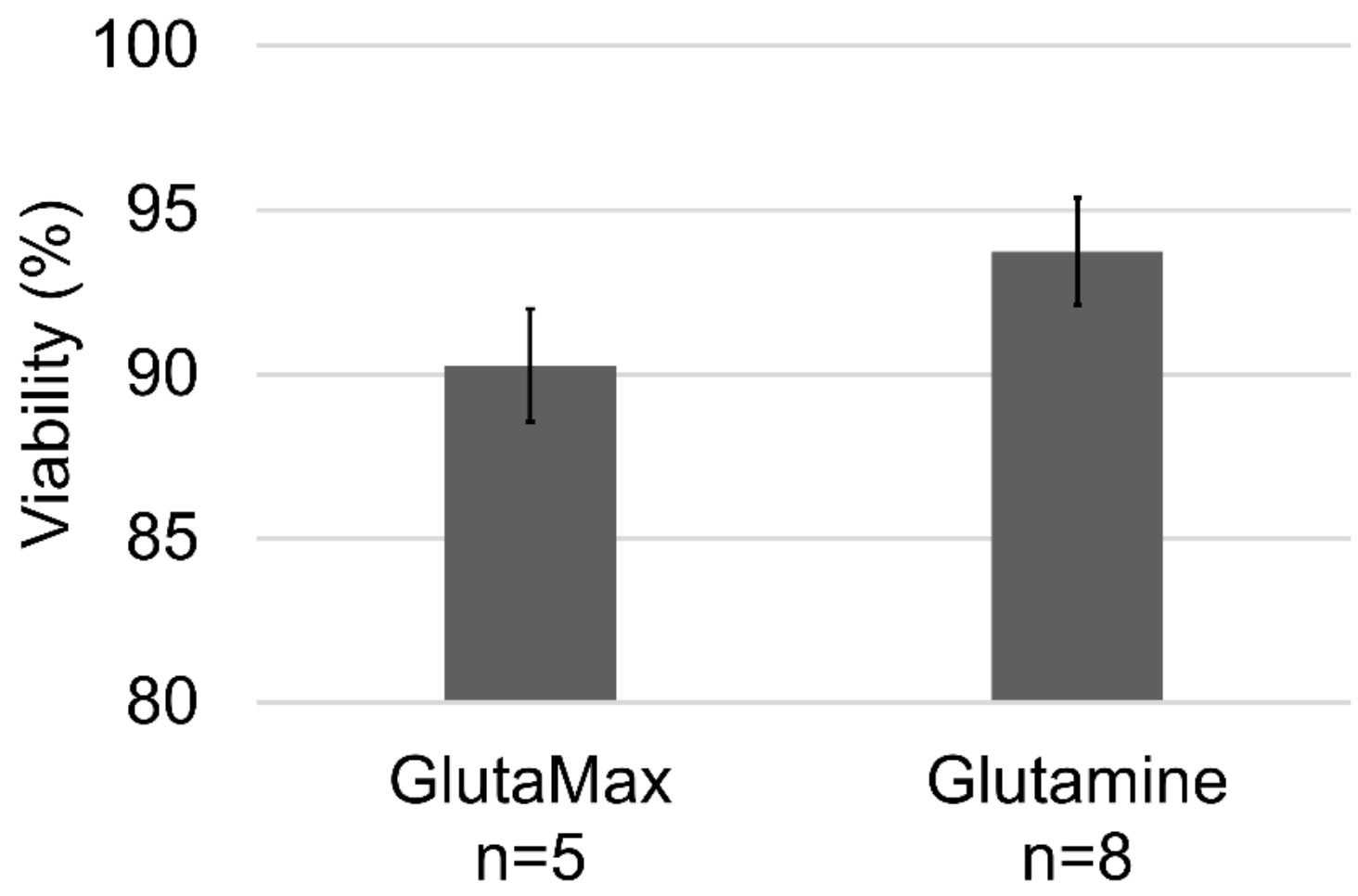

Figure S3 Viability of Vero cells cultured with GlutaMax or glutamine as supplements

Vero cells were cultured with VP-SFM medium in T flasks, and GlutaMax (n=5) or glutamine (n=8) were used as source of glutamine. Viabilities of the cells were shown in the figure.

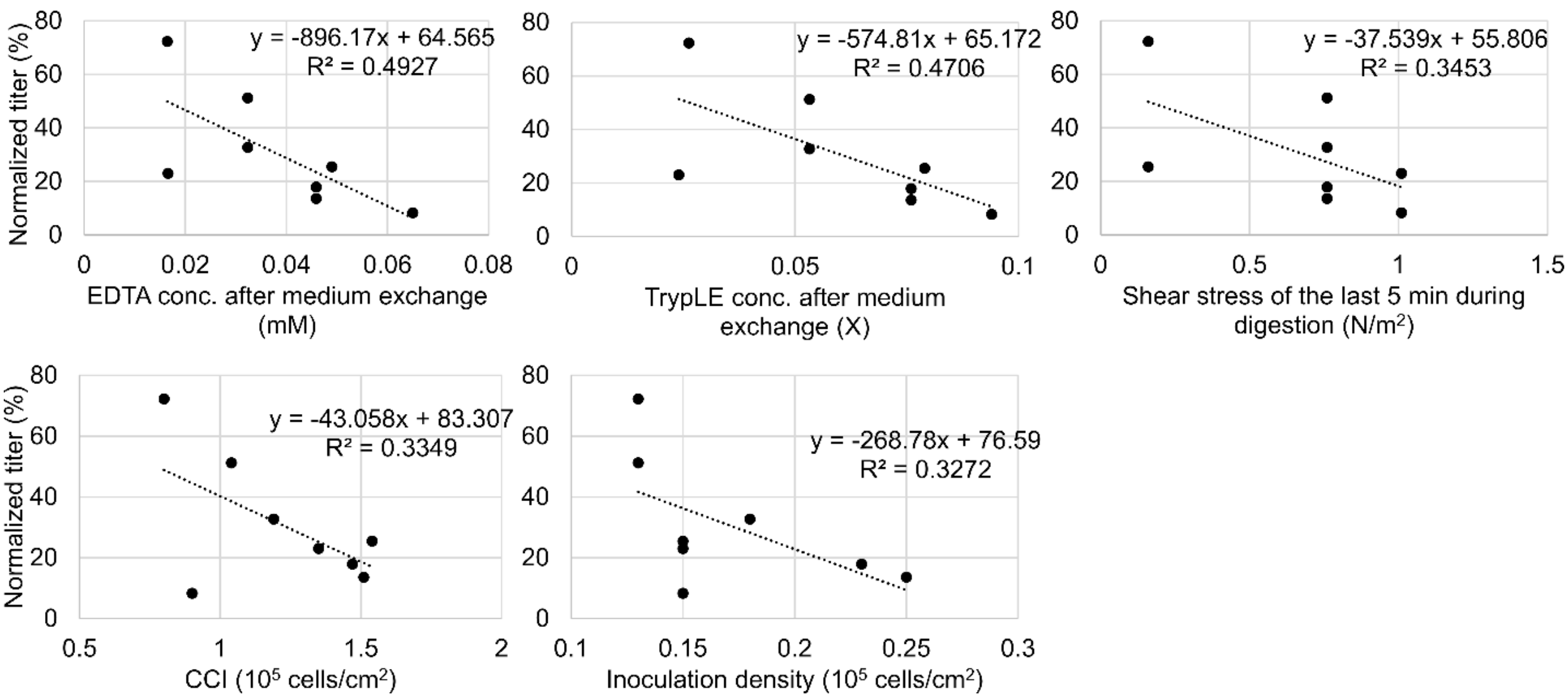

Figure S4 Linear regression analysis between screened operation parameters and normalized titer. Correlation between normalized titer and the five operation parameters (EDTA and TrypLE concentration after medium exchange, shear stress of the last 5 min during digestion, CCI and inoculation density) were indicated by correlation analysis and PCA. And linear regression was performed to select the optimized level of these parameters.

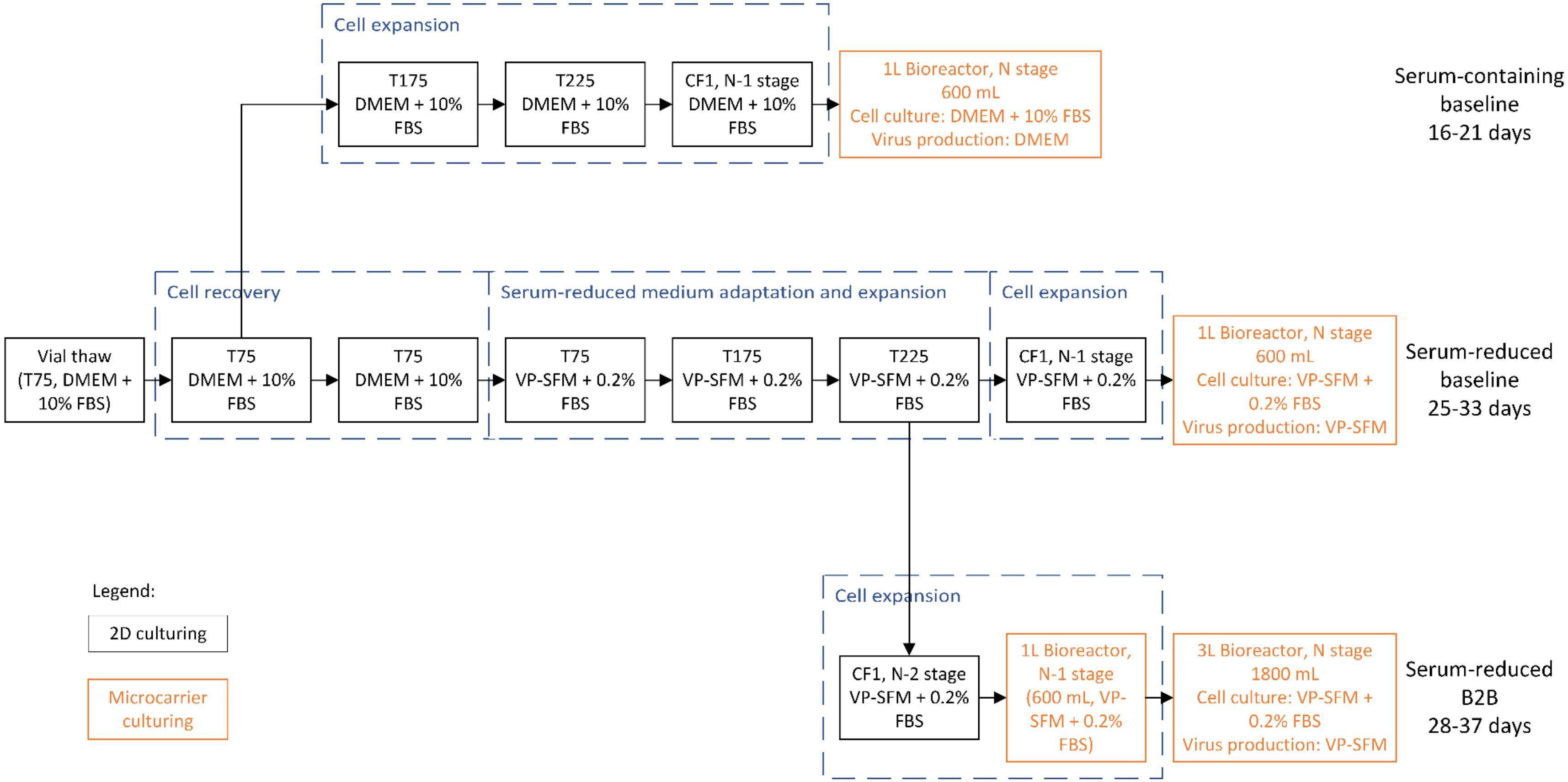

Figure S5 Expansion flow chart for Vero cell culture and HSV-1 production. For the serum-containing baseline process, the cells were recovered and amplified with 2D culturing in DMEM + 10% FBS medium, and finally inoculated into a 1L bioreactor for virus production. For the serum-reduced baseline process, the cells were vial thawed and passaged twice in T75 flask in DMEM + 10% FBS medium for cell recovery, and then directly adapted to VP-SFM + 0.2% FBS medium and amplified simultaneously in 2D culture vessels, then inoculated into a 1L bioreactor for virus production. For the serum reduced bead-to-bead transfer process, the cells cultured in CF1 were inoculated into a 1L bioreactor as N-1 stage. Then bead-to-bead transfer was performed and

the cells were inoculated into a 3L bioreactor for N-stage virus production. Serum was only used during cell expansion, and serum-free medium was used during virus production in N-stage.

Supplementary tables:

Table S1 Experimental design

Category	Evaluated Parameter	Culture Vessel	Evaluated Performance
Medium composition optimization	Basal medium type	T flask	Doubling time, viability, virus titer
	Serum concentration		
	Glutamine supplement type and concentration		
Baseline process transfer from spinner flask to bioreactors	Agitation speed	Bioreactor	Microcarrier uniformity
	Test baseline process in bioreactors	Bioreactor	Cell growth rate, viability, metabolite levels, and virus titer
Preliminary bead-to-bead transfer process development	TrypLE concentration	Spinner flask	Detaching percentage, overall recovery proportion, and viability drop after digestion<br>Cell growth rate, viability, metabolite levels, and virus titer in subculture
	Agitation during digestion		
	Residue volume during washing procedure		
	Transfer operation reduction		
	Cell and microcarrier separation		
	Medium exchange in subculture		
Virus production optimization of the bead-to-bead transfer process	47 parameters from historical experiments	N/A	Correlation with virus titer
	Residue EDTA and TrypLE concentration in subculture	Spinner flask	Detaching percentage, overall recovery proportion, and viability drop after digestion<br>Cell growth rate, viability, metabolite levels, and virus titer in subculture
	Shear stress during agitation		
	Cell densities of inoculation		
	CCI		
Bead-to-bead transfer process in bioreactors	Test optimized bead-to-bead transfer, and subsequent cell culture and virus production processes in bioreactors	1L bioreactor for N-1 stage<br>1L and 3L bioreactors for N-stage	Detaching percentage, overall recovery proportion, and viability drop after digestion<br>Cell growth rate, viability, metabolite levels, and virus titer in subculture

Table S2 Performance and fluid dynamics parameters measured or calculated during the whole procedure

Process stage	Measured or calculated performance parameters
Vial thaw and cell amplification with 2D culturing	Measured: VIA and VCD during cell passage<br>Calculated: specific growth rate
N-1 cell amplification with microcarrier	Measured: daily sampling and measure of VIA, VCD, osmolality, pH, pCO_2, pO_2, concentrations of Na^+, K^+, glucose, ammonium, lactose, glutamine, and glutamic acid.<br>Calculated: specific growth rate
Bead-to-bead transfer	Measured: VIA and VCD before the microcarrier sedimentation of wash ("before wash" for short), before digestion, and before inoculation<br>Calculated: viability drop, overall recovery proportion, and calibrated overall recovery proportion.<br>$$Viability\ drop = VIA_{before\ wash} - VIA_{before\ inoculation}$$<br>$$Overall\ recovery\ proportion = \frac{VCD_{inoculum} \times Vol_{inoculum}}{VCD_{before\ wash} \times Vol_{before\ wash}} \times 100\%$$<br>$$Overall\ recovery\ proportion_{Calibrated} = \frac{VCD_{inoculum} \times Vol_{inoculum}}{VCD_{before\ wash} \times Vol_{before\ wash} - Sampling\ loss} \times 100\%\ *$$<br>Sampling loss was calculated as followed:<br>$$Sampling\ loss = VCD_{Sample\ 1} \times Vol_{Sample\ 1} + VCD_{Sample\ 2} \times Vol_{Sample\ 2} + \cdots + VCD_{Sample\ n} \times Vol_{Sample\ n}$$<br>* Calibrated overall recovery proportions were only used in experiments conducted with spinner flasks. The overall recovery proportions in bioreactors were not calibrated as sampling loss was neglectable.
N-stage cell culturing	Measured: daily sampling and measurement of VIA, VCD, osmolality, pH, pCO_2, pO_2, concentrations of Na^+, K^+, glucose, ammonium, lactose, glutamine, and glutamic acid.<br>Calculated: specific growth rate
Virus infection and amplification	Measured: daily sampling and measurement of VIA, VCD, osmolality, pH, pCO_2, pO_2, concentrations of Na^+, K^+, glucose, ammonium, lactose, glutamine, and glutamic acid; sampling and measurement of volumetric virus titers at different HPIs.<br>Calculated: specific growth rate, cell-specific virus production, normalized volumetric titer
Fluid dynamics parameters	Power input per volume:<br>$$\mathrm{P/V} = N_p n^3 \rho D_i^5 / V$$

Process stage	Measured or calculated performance parameters
	Kolmogorov eddy length: $$\eta = \left[v^3/\left(N_p n^3 D_i^5/V\right)\right]^{\frac{1}{4}}$$ Maximum shear stress: $$\tau_{max} = 5.33\rho\left(vN_p n^3 D_i^5/V\right)^{\frac{1}{2}}$$ η is Kolmogorov eddy size (m), v is kinematic viscosity (m^2/s), V is volume (m^3), N_p is impeller's Newton number or impeller power number, n is agitation speed (r/s), ρ is culture medium density (kg/m^3), D_i is impeller diameter (m).

Table S3 Analyzed operation and performance parameters

Process stage	Parameter	Short name	Type	Tested range	Unit
N-1 culturing	Transfer VCD - N-1 stage	Transfer_VCD	Process parameter	1.56 - 1.93	10^5 cells/cm^2
N-1 culturing	VIA before digestion - N-1 stage	N-1_VIA_before_digestion	Performance parameter	99.1 - 99.2	%
Digestion	TrypLE exposure time	Tryp_exposure_time	Process parameter	54 - 76	min
Digestion	Shear stress of the beginning 30/35 min during digestion	Shear35	Process parameter	0.16 - 0.19	Pa
Digestion	Shear stress of the last 5 min during digestion	Shear5	Process parameter	0.16 - 1.01	Pa
Digestion	Cell/microcarrier separation	MC_cell_sep	Process parameter	Yes/no	N/A
Digestion	VIA after digestion - N-1 stage	VIA_post_digestion	Performance parameter	95.5 - 98.2	%
Termination	TrypLE concentration after termination	Tryp_post_ter	Mixed	0.74 - 1.58	X
Termination	EDTA concentration after termination	EDTA_post_ter	Mixed	0.51 - 0.96	mM
Termination	Medium proportion after termination	Med_prop_post_ter	Mixed	0 - 39.4	%
N-stage inoculation	Inoculation VCD - N-stage	Ino_VCD	Process parameter	0.13 - 0.25	10^5 cells/cm^2

Process stage	Parameter	Short name	Type	Tested range	Unit
N-stage inoculation	Inoculum volume - N-stage	Seed_vol	Mixed	2.37 - 15.5	mL
N-stage inoculation	Calculated trypLE concentration after inoculation	Try_post_ino	Process parameter	0.026 - 0.30	X
N-stage inoculation	Calculated EDTA concentration after inoculation	EDTA_post_ino	Process parameter	0.016 - 0.18	mM
N-stage inoculation	VIA upon inoculation	VIA_upon_ino	Performance parameter	96.8 - 100	%
N-stage cell culturing	Medium exchange after attaching period	Ex_post_att	Process parameter	Yes/no	N/A
N-stage cell culturing	TrypLE concentration after medium exchange	Tryp_post_ex	Process parameter	0.024 - 0.094	X
N-stage cell culturing	EDTA concentration after medium exchange	EDTA_post_ex	Process parameter	0.016 - 0.065	mM
N-stage cell culturing	VCD one day before infection	VCD_1d_before_inf	Performance parameter	0.45 - 0.94	10^5 cells/cm^2
N-stage cell culturing	VIA one day before infection	VIA_1d_before_inf	Performance parameter	96.7 - 99.7	%
N-stage cell culturing	VIA right before infection	VIA_upon_inf	Performance parameter	99.3 - 100	%
N-stage cell culturing	Cell specific growth rate of day 1 - N stage	GR_day1	Performance parameter	0.18 - 1.35	day^{-1}
N-stage cell culturing	Cell specific growth rate of day 2 - N stage	GR_day2	Performance parameter	0.62 - 1.21	day^{-1}
N-stage cell culturing	Cell specific growth rate of day 3 - N stage	GR_day3	Performance parameter	0.16 - 0.68	day^{-1}
N-stage cell culturing	Glucose concentration one day before infection	Gluc_1d_before_inf	Performance parameter	2.4 - 3.5	g/L
N-stage cell culturing	Glucose concentration right before infection	Gluc_before_inf	Mixed	1.25 - 2.03	g/L

Process stage	Parameter	Short name	Type	Tested range	Unit
Virus production	CCI	CCI	Process parameter	0.80 - 1.54	10^5 cells/cm^2
Virus production	VCD one day after infection	VCD_1d_post_inf	Performance parameter	0.49 - 1.71	10^5 cells/cm^2
Virus production	VCD two days after infection	VCD_2d_post_inf	Performance parameter	0.27 - 0.86	10^5 cells/cm^2
Virus production	VIA one day after infection	VIA_1d_post_inf	Performance parameter	74.5 - 99.6	%
Virus production	VIA two days after infection	VIA_2d_post_inf	Performance parameter	59.0 - 82.4	%
Virus production	Cell specific growth rate of the first day after infection - N stage	GR_1d_post_inf	Performance parameter	-0.58 - 0.61	day^{-1}
Virus production	Cell specific growth rate of the second day after infection - N stage	GR_2d_post_inf	Performance parameter	-1.22 - -0.59	day^{-1}
Virus production	Glucose concentration one day after infection	Gluc_1d_post_inf	Mixed	2.59 - 3.73	g/L
Virus production	Glucose concentration two days after infection	Gluc_2d_post_inf	Mixed	1.81 - 3.44	g/L
Virus production	Lactate concentration one day after infection	Lac_1d_post_inf	Performance parameter	0.81 - 1.73	g/L
Virus production	Lactate concentration two days after infection	Lac_2d_post_inf	Performance parameter	1.07 - 2.53	g/L
Virus production	Ammonia concentration one day after infection	Amo_1d_post_inf	Performance parameter	1.73 - 2.64	g/L
Virus production	Ammonia concentration two days after infection	Amo_2d_post_inf	Performance parameter	2.35 - 3.00	mM
Virus production	Glutamine concentration one day after infection	Gln_1d_post_inf	Performance parameter	1.22 - 2.13	mM
Virus production	Glutamine concentration two days after infection	Gln_2d_post_inf	Performance parameter	0.81 - 1.21	mM

Process stage	Parameter	Short name	Type	Tested range	Unit
Virus production	Normalized titer: ratio of sample/internal control infectivity titer	Titer_sample_vs_intctrl	Performance parameter	8.30 - 72.3	%

Table S4 Marks of secondary correlation analysis

Parameters	Negative mark	Positive mark	Total mark
Transfer_VCD	0	3	3
Tryp_exposure_time	0	0	0
Shear35	-4	1	-3
Shear5	-7	0	-7
MC_cell_sep	-1	0	-1
Ino_VCD	-4	0	-4
Ex_post_att	-2	3	1
Tryp_post_ex	-10	0	-10
EDTA_post_ex	-10	0	-10
CCI	-4	0	-4

Table S5 Levels of selected operation parameters in original and optimized processes

Parameter	Original level[*1]	Optimized level[*2]
EDTA concentration after medium exchange (mM)	<0.64	<0.16
TrypLE concentration after medium exchange (X)	<0.08	<0.02
Maximum shear stress of last 5 min during digestion (N/m^2)	1.02	0.38
CCI (10^5 cells/cm^2)	1.5±0.2	1.1±0.2
Inoculation density (10^5 cells/cm^2)	0.15±0.3	0.12±0.3

*1 Average level of historical data.

*2 Theoretical level for process design. Applied level may fluctuate around 20% due to operation deviations.

Table S6 Abbreviations

Abbreviation	Full name
B2B	Bead-to-bead transfer
BM	Basal medium
BR	Bioreactor
CCI	Cell concentration at the point of infection
CF	Cell factory
DMEM	Dulbecco's modified eagle medium
DO	Dissolved oxygen
DPBS	Dulbecco's phosphate-buffered saline
FBS	Fetal bovine serum
HPI	Hour post-infection
HSV-1	Herpes simplex virus type-1
JS	Just suspended
OV	Oncolytic virus
PCA	Principal component analysis
rpm	Revolutions per minute
SCM	Serum-containing medium
SP	Spinner flask
SRM	Serum-reduced medium
STI	Soybean trypsin inhibitor
VCD	Viable cell density
VIA	Viability
